# Smartphone-based digital markers and clinical symptoms during therapy for Borderline Personality Disorder

**DOI:** 10.1016/j.invent.2026.100952

**Published:** 2026-05-25

**Authors:** Ana Macchia, Dimitri Löchner, Ann-Christin Haag, Christopher Kannen, Christian Montag, Birgit Abler

**Affiliations:** a Ulm University, Clinic for Psychiatry/Psychotherapy III, Germany; b Institute of Psychology, University of Tartu, Tartu, Estonia; c Ulm University, Department of Child and Adolescent Psychiatry/ Psychotherapy, Germany; d Ulm University, Department of Department of Molecular Psychology, Germany; e Centre for Cognitive and Brain Sciences, Institute of Collaborative Innovation, University of Macau, Macau; f Department of Psychology, Faculty of Social Sciences, University of Macau, Macau; g Department of Computer and Information Science, Faculty of Science and Technology, University of Macau, Macau

**Keywords:** Digital phenotyping, Smartphone usage behavior, Dialectic behavior therapy (DBT), Borderline Personality Disorder (BPD)

## Abstract

No established digital markers yet exist for borderline personality disorder (BPD) or for predicting symptom change throughout therapy. This study adopts an exploratory approach to uncover candidate associations between smartphone behavior and BPD symptoms.

We objectively recorded smartphone use in individuals with BPD (*N* = 49) during an eight-week inpatient DBT program. Symptom severity was assessed using the Borderline Symptom List (BSL-23) pre- and post-DBT, and the Dissociative Experiences Scale (DES-II) at baseline. Smartphone usage time and communication behavior (phone calls, SMS) were compared with *t*-tests to student samples. We explored Spearman correlations between smartphone usage and pre- and post-therapy BSL, DES-II as well as BSL symptom change. To predict treatment outcomes by smartphone variables, we modeled a multiple linear regression controlling for age, baseline symptom severity, and pretreatments.

Compared to student samples, individuals with BPD used their smartphones more than twice as long (5.88 h per day) and made a higher number of phone calls. Higher average smartphone usage time was associated with a greater trait tendency to dissociate (DES-II), whereas no other digital markers were linked to baseline BPD symptoms. Locked screen time was linked to poorer symptom improvement, while a higher number of incoming calls was associated with better treatment outcomes. These effects already emerged in the first week smartphone use and explained 60% of the variance.

Excessive smartphone use and call behavior may function as digital markers of BPD, with more dissociative patients showing particularly high use. Higher locked screen time, potentially reflecting maladaptive checking behavior, was associated with poorer treatment outcomes, whereas a greater number of incoming calls, possibly indicative of social support, was associated with better improvement. These findings suggest that digital phenotyping may help to identify early markers of recovery when integrated into psychotherapy. However, due to the small sample size, the unmatched student control group, and the exploratory nature of the analyses, the results of the study should be interpreted with caution, and their generalizability should be examined in future studies.

**Trial registration:**

retrospectively registered - https://osf.io/dfq9y/?view_only=4c19b891bb6448009b22f60b2552bd73

## Highlights

• Smartphone use in BPD exceeded student samples by more than twofold.
• Greater smartphone use was associated with higher trait dissociation.
• Locked screen time predicted poorer symptom improvement during therapy.
• Incoming calls were associated with better clinical outcomes.
• Digital phenotyping may support early intervention in psychiatry.

## 1 Introduction

Dysfunctional management of emotions, identity disturbances, and interpersonal challenges are core deficits in patients with borderline personality disorder (BPD; Richetin et al., 2017). These symptoms can fluctuate markedly within hours or days, posing challenges for reliable assessment. Traditional approaches relying on retrospective self-reports provide only coarse, episodic snapshots of patients' experiences and are prone to recall bias (Paulhus and Vazire, 2007). In contrast, digital behavioral markers derived from smartphone usage patterns, such as overall usage time and call or message frequency, offer the opportunity to capture behavior in a continuous and ecologically valid manner to gain more insights in psychiatric disorders (Hidalgo-Mazzei et al., 2018; Rozgonjuk et al., 2023). This may be a particularly promising approach in BPD, as personality traits have been shown to relate to smartphone usage patterns, and digital communication might serve as a mean to cope with attachment anxiety – a core feature of BPD (Konok et al., 2016; Marengo et al., 2023; Stachl et al., 2017). Moreover, difficulties in emotion regulation, another core feature of BPD, appear to be closely related to smartphone use behavior (Rozgonjuk and Elhai, 2021). However, a better understanding of how passively collected digital markers relate to self-reported symptoms is crucial for validating their relevance for actual clinical practice (Fulford and Jacobson, 2025), elucidating real-world behavioral mechanisms of BPD, and laying the groundwork for future integration into evidence-based psychiatric assessment. Smartphone-based digital markers may be particularly relevant for symptoms that are difficult to capture, such as dissociative experiences, which are often transient and easily overlooked in conventional assessments (Al-Shamali et al., 2022). At the same time, current debates in digital psychiatry emphasize that such data require cautious interpretation, as they are context-dependent and not inherently indicative of specific clinical processes (Beg et al., 2025; Beg and Verma, 2025).

Disorder-specific psychotherapeutic interventions are the gold standard for the treatment of patients with BPD, with dialectical behavior therapy (DBT) being one of the most established and empirically supported approaches (Stoffers-Winterling et al., 2012). Even though, psychotherapeutic interventions have been shown to be effective, outcomes vary widely, and reliable predictors of treatment response are scarce, especially in naturalistic settings (Herzog et al., 2020; Stoffers-Winterling and Lieb, 2022). So far, only few well-established predictors of naturalistic DBT treatment outcomes exist, and these often involve stable factors such as age or previous treatment history that cannot be modified (Herzog et al., 2020; Mikusky et al., 2025). Smartphone-based digital markers may provide early insights into behavioral processes predictive of treatment outcomes. By identifying modifiable behavioral patterns, they hold promise for more personalized and dynamic approaches in BPD care.

A recent systematic review suggests that digital phenotyping is already a promising approach in mood disorders, schizophrenia, and anxiety disorders, where features such as mobility, sleep, and communication patterns relate to psychiatric symptoms (Bufano et al., 2023). In depression, smartphone interaction patterns, including screen on/off behavior and call behavior, have been proposed as relevant indicators of depressive symptom severity and symptom change (Ikäheimonen et al., 2024; MacLeod et al., 2021; Opoku Asare et al., 2021). Another study in individuals recruited from a university setting distinguishes smartphone checking behavior from overall screen time and shows that the frequency of checks, rather than total usage duration, is associated with a higher rate of daily cognitive failures referring to momentary lapses in attention, memory, or action – such as forgetting, or getting distracted (Hartanto et al., 2023). In a student sample, individuals with higher levels of depressive symptoms unlocked their phones less frequently, which may be related to avoidance behavior or social isolation (Rozgonjuk et al., 2018). However, potential smartphone-based digital markers in BPD remain scarce. A study by Gillett et al. (2021) with a small sample of BPD individuals (*n* = 17), bipolar disorder (*n* = 17), and healthy controls (*n* = 21) showed that transdiagnostically, elevated mood was linked to increased call and text activity, whereas depressive symptoms were associated with longer incoming call duration. On the diagnostic level, BPD exhibited increased frequency and length of outgoing text messages compared to healthy controls, suggesting that communication dynamics may be relevant digital markers in BPD.

### 1.1 Study aim and hypotheses

The present study investigates passively tracked smartphone data from individuals with BPD undergoing an 8-to-10-weeks inpatient DBT program to identify smartphone-based digital markers reflecting borderline-specific patterns of smartphone use, compared to a student sample, and borderline symptom severity, as well as to explore potential digital behavioral predictors of psychotherapeutic treatment outcome. In doing so, it aims to bridge the gap between traditional diagnostic self-assessments and continuous, passive, smartphone-based real-world monitoring, which may pave the way for developing digital markers that serve as early warning signals or real-time indicators of therapeutic change. We hypothesized that smartphone usage patterns would be linked to BPD psychopathology and suited to predict symptom change throughout therapy; however, as almost no prior studies have examined specific associations between smartphone usage patterns and symptom markers, this approach was inherently exploratory and aimed at identifying potential markers rather than testing predefined hypotheses.

## 2 Methods

### 2.1 Sample

Participants were recruited from the inpatient DBT program of the Clinic for Psychiatry/Psychotherapy III, Ulm University, Germany between April 2022 and June 2023. The initial study sample comprises a subsample of a larger study evaluating outcomes of inpatient DBT in a naturalistic context (Mikusky et al., 2025) and consisted of 59 participants who underwent a planned 8 to 10-week therapy program. The Ethics Committee of Ulm University (EA 301/21) approved the study protocol.

All patients who agreed to have their smartphone use monitored by the *Insights* app provided written informed consent in accordance with the Declaration of Helsinki and ethical guidelines. The *Insights* app is an Android-based research tool that passively tracks smartphone usage behavior and actively collects self-report data through customizable questionnaires, enabling researchers to study human behavior in everyday life (Messner et al., 2019; Montag et al., 2019).

We included data from all participants of the DBT program who (1) were Android-users and chose digital monitoring on their own smartphone using the *Insights* app instead of the paper-based version during their inpatient stay, (2) had a BPD diagnosis confirmed by a senior physician using the International Personality Disorder Examination for ICD-10 (IPDE; Loranger et al., 1997), and (3) whose devices recorded smartphone use for at least three days. The app was only available for Android systems.

The effective sample consisted of 49 participants with BPD, mainly young and female (four male, 44 female, one transgender female-to-male; age 18 to 44 years; *M* = 26.53 years, *SD* = 7.31 years). 47 of the participants were diagnosed with emotionally unstable personality disorder, borderline type and two with emotionally unstable personality disorder of the impulsive type. The median number of comorbid diagnoses was two with major depressive disorder (single episode or recurrent, *n* = 32, 65%) and posttraumatic stress disorder (*n* = 20, 41%) as the most common comorbid diagnoses.

On average inpatient stays lasted 53 days (*SD* = 15.34). Seven of the 49 patients included in the study discontinued therapy before completing the DBT program, on average after 32 treatment days (*SD* = 19.36). The *Insights* app tracked on average 37.63 treatment days (*SD* = 17.1, *MDN* = 37, range: 4–56). There was no financial compensation for study participation. For reference, published mean data from university students not matched to the patient sample that were investigated in 2017 and 2018 in two previous studies using the *Insights* app conducted at Ulm University could be obtained (Messner et al., 2019; Montag et al., 2019).

### 2.2 Instruments

To capture digital behavior in real-life contexts, the *Insights* app was used, which allows for continuous collection of a wide range of smartphone-based behavioral data. A detailed description of the app and its applications is provided elsewhere (Montag et al., 2019). At the beginning of the study participation, the *Insights* app was installed on the participants' own smartphone device, usually on the day after admission to the program. At the end of the inpatient stay, participants were instructed to uninstall the *Insights* App from their smartphones. This study aims to explore overall smartphone usage as well as usage patterns, especially communication behavior, among individuals with BPD and their relation to symptoms (https://osf.io/dfq9y/?view_only=4c19b891bb6448009b22f60b2552bd73; Hypothesis III). Additional smartphone variables (e.g. battery status) were collected but are not further described in the present study. Of special relevance to this study, the *Insights* app recorded digital markers regarding communication behavior (number of incoming and outgoing calls, number of incoming and outgoing Short Message Service (SMS)), the smartphone usage time (i.e., any user interaction in an unlocked state, including app use, communication, system interactions, and other foreground processes), the smartphone usage frequency (i.e., count of individual active app sessions), and the locked screen time (i.e., any user interaction in a locked state, including checking behavior of notifications, time, messages and quick-access features available on the lock screen such as the camera or flashlight). As the number of incoming and outgoing SMS messages was low and we did not include text messages from other commonly used messenger services such as WhatsApp, a combined total SMS score was calculated. Data were first aggregated on a daily level and then averaged across the entire treatment period, adjusted for each participant's individual tracking duration.

To assess BPD symptoms in the past week at admission and discharge, we included the short version of the German Borderline Symptom List (BSL-23; Bohus et al., 2009), which comprises 23 items. The BSL-23 demonstrated excellent internal consistency in our sample (Macchia et al., 2026a). Furthermore, participants answered the German adaption of the Dissociative Experiences Scale (DES-II; Freyberger et al., 1998) at baseline, to measure trait dissociation.

### 2.3 Statistical analyses

Data processing and statistical analyses were performed using Rstudio Version 4.3.1 (R Core Team, 2023). To compare our data with those from previous studies, we used Welch's two-sample *t*-test, which does not assume equal variances between groups. As our data were additionally not normally distributed, we computed bootstrapped estimates of Cohen's *d* (5,000 resamples) to obtain a robust measure of effect size and corresponding confidence intervals. To explore whether smartphone communication (incoming/outgoing phone calls, total SMS), usage time and frequency, such as locked screen time are associated with Borderline Symptoms (BSL-23), we calculated Spearman correlations for the BSL pre-therapy and post-therapy such as symptom change during therapy (BSL difference score: BSL-23 post therapy – BSL-23 pre therapy). Additionally, we explored the association between smartphone variables and trait dissociation assessed at baseline (DES-II).

Since our exploratory analyses indicated associations between smartphone usage patterns and changes in BSL symptoms over the course of therapy, we calculated a linear model using smartphone usage averaged across the entire treatment period, as well as an additional model that included only the smartphone tracking data from the first seven days of DBT. The models included the BSL difference score as outcome variable as correlate of therapeutic success. The BSL pre-therapy score, locked screen time, the number of incoming calls, such as age, and the number of previous inpatient treatments (at least one week) were included as predictor variables. Hereby, only participants who stayed for at least three weeks (*N* = 46) were included. This model accounts for the fact that baseline symptom severity and pretreatment psychopathology typically predict the magnitude of symptom change during psychotherapy (Barnicot et al., 2012; Herzog et al., 2020) and controls for age, given its known association with smartphone use patterns (Csibi et al., 2021). All variables were z-standardized prior to analysis. Multiple imputation was used to reduce bias associated with missing data due to dropout, as it is common in psychotherapy research. Compared to complete-case analyses, this approach makes use of all available data and might yield more efficient and less biased estimates, and is therefore recommended over leaving missing data untreated (Moore, 2024; Van Buuren, 2018). Missing post-treatment BSL values (*N* = 9) were imputed using multiple imputation by chained equations (MICE) with *m* = 20 imputations and up to 10 iterations. The final “best-guess” estimate for each missing case was obtained as the mean across all imputed datasets. We applied 1000 resamples of bootstrapping on beta estimates to proof the robustness (Mason et al., 2021) of our linear models and checked each model for testing assumptions including outliers with cook's distance. To further assess robustness, we conducted additional analyses without multiple imputation and control variables, which identified the same predictors as meaningful. We did not apply corrections for multiple comparisons because the analyses were exploratory and hypothesis-generating, intending to identify potential patterns of associations suited to motivate further research.

## 3 Results

### 3.1 Smartphone use in borderline personality disorder and university students

The samples of university students in Montag et al.'s (2019) study applied for exploratory comparisons were significantly younger, and included a higher proportion of male participants compared to our sample (Table 1). Descriptive data shows that individuals with BPD use their smartphones during inpatient therapy on average for 352.66 min (5.88 h) per day, which is more than twice as long as university students. This group difference was supported by a very large effect size (*d* = −1.58). Moreover, individuals with BPD tended to make and receive phone calls more frequently than students. Yet, this effect should be interpreted cautiously, as indicated by the bootstrapped effect sizes (see Table 1), and no differences emerged regarding SMS. Table 1 displays the mean daily smartphone usage metrics of our sample in comparison to reference values from the student samples collected in previous *Insights* App studies (Messner et al., 2019; Montag et al., 2019).

**Table 1:** Daily smartphone usage metrices of BPD patients and university students.

	BPD		C				
	*M/N*	*SD/%*	*M/N*	*SD/%*	*t/X*^*2*^	*Cohens d* [95% BCa CI]^a^	*p*
Age (Messner et al., 2019)	26.53	7.31	22.40	6.10	−3.58	−0.64 [−0.64, −0.97]	<0.01⁎⁎⁎
Gender (Messner et al., 2019)	44	89.8	115	72.3	5.41	0.16 [0.07, 5.15]	0.020⁎
Age (Montag et al., 2019)	26.53	7.31	23.34	7.59	−2.50	−0.43 [−0.77, −0.08]	0.014⁎⁎
Gender (Montag et al., 2019)	44	89.8	69	65.1	9.14	0.24 [0.11, 7.75]	0.003⁎⁎
Usage time (min/day)	352.66	205.63	168.77	98.52	−6.05^a^	−1.58 [−1.99, −1.17]	<0.001⁎⁎⁎
Usage frequency (nr/day)	287.99	184.31	*NA*	*NA*	*NA*	*NA*	*NA*
Locked screen time (min/day)	103.16	126.17	*NA*	*NA*	*NA*	*NA*	*NA*
Incoming calls (nr/day)	0.98	1.11	0.45	0.49	−3.20	−0.52 [−0.94, −0.09]	0.002⁎⁎
Outgoing calls (nr/day)	2.57	3.44	1.42	1.77	−2.21	−0.27 [−0.67, 0.13]	0.031⁎
Total SMS (nr/day)	0.77	1.00	0.64	1.04	−0.79	0.14 [−0.18, 0.45]	0.43 *ns*

*Note.* BPD: Borderline personality disorder (N = 49). C: Control participants (university students) from Montag et al., 2019 (*N* = 106) and Messner et al., 2019 (*N* = 157). Gender is reported as the number of women relative to men and transgender participants. Min = minutes. Nr = number. Usage time: Average daily smartphone usage time per day in minutes. Usage frequency: Average daily number of smartphone usage frequency (i.e., count of individual active app sessions). Locked screen time: Average time using the smartphone without unlocking it per day. Incoming/outgoing calls: Average number of incoming or outgoing calls per day. Total SMS: Total average number of received and sent text messages per day.

a Cramér's V was included as an effect size for frequency data. Values of approximately 0.10, 0.30, and 0.50 may be interpreted as small, medium, and large effects, respectively.

⁎ Indicates *p* < .05.

⁎⁎ Indicates *p* < .01.

⁎⁎⁎ Indicates *p* < .001.

### 3.2 Smartphone-based digital markers and borderline symptom severity

**Fig. 1:**
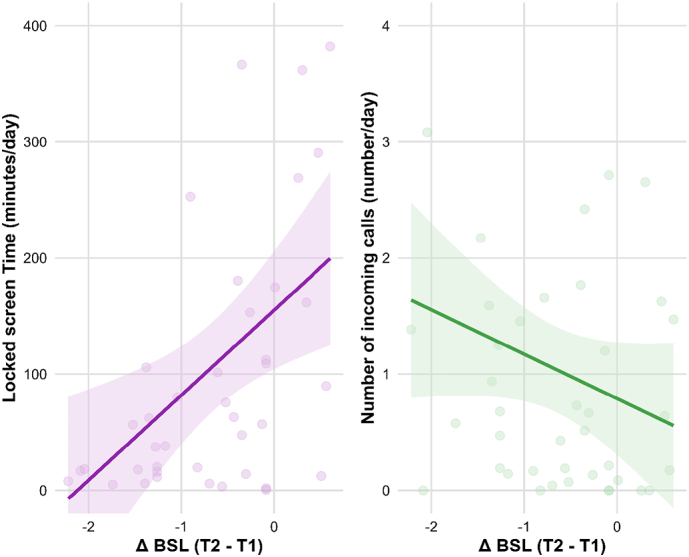
Associations between smartphone-based digital markers and symptom change during DBT.

No associations emerged between any digital marker and baseline BSL scores. Higher trait dissociation at baseline (DES-II) related to higher smartphone usage duration per day, *r*ₛ = 0.34, 95%CI [0.03; 0.62], *p* = .031. At discharge, patients who received a higher number of incoming phone calls during therapy showed lower BSL scores, *r*ₛ = −0.38, 95%CI [−0.60; −0.03], *p* = .047 (Fig. 1), whereas no other digital indicator correlated with post-treatment borderline symptom severity. Less improvement in BSL scores over the course of therapy was linked to longer locked screen time, *r*ₛ = 0.55, 95%CI [0.21; 0.80], *p* < .001 (Fig. 1), while all other markers showed no significant relationships.

The left panel depicts the association between average daily locked-screen time and BSL symptom change (T2: BSL-23 post therapy – T1: BSL-23 pre therapy), indicating that greater locked-screen time was related to less symptom improvement during DBT. The right panel shows the association between the average number of incoming phone calls per day and BSL symptom change, suggesting that a higher number of incoming calls tended to relate to a greater symptom improvement. Note that few data points may overlap or fall outside the visible range of the figure.

### 3.3 Smartphone-based digital markers and symptom change during psychotherapy

A multiple linear regression model was conducted to examine variables of symptom change during DBT (T2: BSL-23 post therapy – T1: BSL-23 pre therapy) using smartphone usage averaged across the entire treatment period, as well as an additional model that included only the smartphone tracking data from the first seven days of the inpatient stay to investigate the explained variance of early therapy usage patterns for outcomes. The overall model for the entire treatment period was significant and explained a substantial proportion of variance in symptom change (*R*^2^ = 0.57). Greater locked screen time was associated with lower symptom improvement (β = 0.41), reflecting a moderate effect. A higher number of incoming phone calls was related to greater symptom improvement (β = −0.29), with a small effect size. The baseline BSL score (T1) strongly related to greater symptom improvement (β = −0.59), whereas age (β = −0.17) and the number of pretreatments (β = −0.10) did not significantly contribute to the model. The model for the first 7 treatment days explained even a higher proportion of variance in symptom change (*R*^2^ = 0.60). As in the model using averaged values across the entire treatment period, baseline BSL scores, locked screen time, and incoming calls emerged as significant variables. Locked screen time (β = 0.38) and incoming calls (β = −0.34) showed almost equally strong average effects—locked screen time was positively associated with symptom change, whereas incoming calls were negatively associated. Table 2 shows details about the multiple linear regression model for the whole treatment period. The model without imputed missing data revealed similar results.

**Table 2:** BSL difference score (BSL T2 - BSL T1) predicted by locked screen time, incoming phone calls, baseline BSL (T1), age, and the number of pretreatments

Predictors	Estimates	CI	p
Intercept	0.02	−0.22–0.26	0.851
Locked screen time	0.41	0.18–0.64	**0.001**
BSL baseline score (T1)	−0.59	−0.84 – -0.34	**<0.001**
Incoming calls	−0.29	−0.53 – -0.05	**0.019**
Age	−0.17	−0.44–0.09	0.193
Number of inpatient pretreatments	−0.10	−0.34–0.13	0.386
Observations	39		
R^2^ / R^2^ adjusted	0.566 / 0.500		

*Note.* Locked screen Time: Average time using the smartphone without unlocking it per day. Incoming calls: Average number of incoming calls per day. Number of inpatient pretreatments: Number of inpatient pretreatments for at least one week.

## 4 Discussion

This study aimed to identify potential digital markers of borderline symptom severity and symptom change during psychotherapy, adopting an exploratory approach to uncover candidate associations in the absence of prior evidence. It advances the field by showing that individuals with BPD display increased smartphone usage time and call frequency compared to student samples. While no digital markers were identified that reflected baseline BPD symptom severity, greater overall smartphone use appeared to characterize BPD individuals with stronger dissociative tendencies. Moreover, less symptomatic improvement during DBT (BSL-23) was related to prolonged locked screen duration, whereas more frequent incoming calls were linked to greater symptom reduction.

Even though our student comparison samples were not matched to the BPD group, the difference between samples with a smartphone use twice as high (5.88 h per day more usage) in the patients as in the students could be meaningful and motivate further research. Other sources also indicate that the observation in individuals with BPD points towards a more extensive smartphone use than the general population: For example, subjectively reported daily usage among 16–29-year-olds is approximately 2.63 h (Bitkom e. V., 2024). Moreover, in their mixed-method study ‘Smartphone Use in Germany in 2023,’ Toth, Parry, and Emmer objectively tracked smartphone behavior in a large German adult sample (*n* = 1798) and found that Android users used their devices for an average of 4.7 h per day (Toth et al., 2024) – again significantly lower than the usage duration observed in our BPD group. Taken together, these consistent discrepancies suggest that more excessive smartphone use may represent a potential digital marker of BPD. Although the underlying mechanisms remain speculative, heightened impulsivity, given that smartphones provide immediate stimulation and have been linked to impulsive behavior, as well as the use of smartphones for emotion regulation or seeking interpersonal connection may contribute to this pattern. These findings align with recent evidence showing that emotion-related impulsivity plays a central role in excessive and dysregulated smartphone behavior, as demonstrated by Cho and Kim's (2025) network analysis identifying impulsivity as a key mechanism underlying smartphone overdependence. However, the inpatient setting might also have influenced patients' behavior, as a lack of familiar peers and environment could affect daily ratings and engagement in self-regulatory strategies compared with patients in outpatient settings.

Moreover, in our sample a higher trait tendency to dissociate was related to longer smartphone usage time. Since dissociation relates to more pronounced symptoms in BPD (Jaeger et al., 2017), a potential interpretation is that this might reflect that patients with a higher burden of disease have a higher need for self-regulation using the smartphone. Dissociation on the other hand could prevent patients from interrupting automated usage patterns. An observation of Ward et al. (2017) who demonstrated that even the mere presence of a smartphone reduces available cognitive capacity could point towards a vicious circle, as the authors interpreted the phenomenon as a form of attentional withdrawal or self-disengagement. Thus, smartphone usage might not only be prolonged due to dissociative symptoms calling for regulation or preventing discontinuation of use but could possibly by itself heighten the disconnection from the self and the environment.

More pronounced phone call behavior may represent another digital marker of BPD. This pattern could reflect core borderline symptoms like the difficulty in maintaining stable interpersonal connections – consistent with the notion that individuals with BPD do not generally withdraw from social contact but rather struggle within relationships once they are formed (Di Bartolomeo et al., 2024). Previous research has also shown that individuals with BPD engage more intensively with social media, potentially to satisfy heightened needs for social connectedness (Ooi et al., 2020). Nonetheless, we assessed only phone call behavior rather than interpersonal functioning in our sample; therefore, it is possible that increased calling reflects other factors, such as situational demands related to inpatient treatment (e.g., informing one's social environment), generally higher social integration (e.g., a larger social network), or organizational stressors (e.g., work or financial issues). In contrast to Gillett's (2021) findings, we did not observe increased messaging behavior in our sample; however, our data discussed in this paper only include SMS, while much contemporary communication takes place via apps such as WhatsApp. Future work should therefore incorporate more detailed usage parameters of communication apps to more accurately capture interpersonal digital behavior. Moreover, to test the specificity of pronounced call behavior for BPD a matched psychiatric control group is required.

From a clinician's perspective, the use of smartphone behavioral markers to predict treatment outcomes is particularly interesting. In our data, locked-screen usage emerged as a negative indicator of therapeutic improvement, suggesting that patients who frequently activate their phone for short, potentially superficial interactions tend to show poorer outcomes. This finding was even evident when only including the first seven days of smartphone tracking into the analyses, indicating that the behavior could be used as a potential marker to identify patients with a greater risk for lower improvement already at the beginning of therapy. Elevated locked-screen use may represent maladaptive regulatory processes that interfere with effective therapeutic change. One possible mechanism is that it reflects rather meaningless, maybe habitual and probably not fully intentional checking behavior. This interpretation is supported by Toth et al.'s (2024) study showing that nearly all deliberate smartphone activity occurs after the device is fully unlocked. In adolescence, social media checking was associated with subsequent immediate increases in positive mood (Dreier et al., 2024), which implies a short-term emotion-regulatory effect of checking. Checking behavior may be particularly driven by fear of missing out, which heightens the urge to repeatedly monitor social cues and potential updates (Przybylski et al., 2013). Another study found that fear of missing out was associated with increased daily disruptions from interruptive notifications, suggesting an implicit link to checking behavior (Rozgonjuk et al., 2019). Combined with the smartphone's intermittent reward structure, where unpredictable notifications act as potent reinforcers (Oulasvirta et al., 2012), checking becomes increasingly habitual and difficult to regulate. Over time, this could create a self-perpetuating cycle in which fear of missing out and reward-driven checking mutually amplify each other, fostering dependency-like patterns of smartphone use. These mechanisms could hamper the use of functional self-regulation strategies and thus diminish therapeutic success. However, locked-screen use may also reflect passive information checking (e.g., viewing the time, notifications, or weather) or quick access to functions such as the camera without fully unlocking the device.

Interestingly, the number of incoming phone calls also emerged as an indicator of borderline symptoms change during DBT in our sample with more calls being associated with greater therapeutic success. Although interpersonal interactions can be experienced as stressful and emotionally overwhelming in individuals with BPD (Hepp et al., 2018; Macchia et al., 2026b), receiving contact from others may nevertheless have a beneficial effect by fostering a sense of connectedness and reducing feelings of isolation, factors that are particularly relevant in BPD (Di Bartolomeo et al., 2024). This interpretation aligns with findings showing that higher perceived social support from family, friends, and significant others is associated with better social wellbeing in BPD outpatients (Gull et al., 2022). Moreover, incoming calls may reflect not only perceived but also actual social support, which could positively contribute to therapeutic progress. However, whether and to what extent incoming calls truly capture social support remains an open and intriguing question for future research.

### 4.1 Strengths and limitations

This study applied an innovative approach by employing digital phenotyping across the entire course of psychotherapeutic treatment to capture real-life behavior. This allowed us to incorporate behavioral digital markers that can complement traditional self-report measures, which are known to be vulnerable to recall bias and subjective distortions. The inpatient setting further ensured that participants were exposed to comparable between-person environmental conditions, thereby reducing variability from external influences related to different work schedules or daily routines.

Nevertheless, several limitations must be acknowledged. First, the sample size was small, and we did not include a matched healthy or clinical comparison group, making it difficult to determine whether the observed digital markers are specific to BPD. The contrast with the control groups is limited by a non-alignment of sociodemographics nor screening for BDP. It is worth emphasizing that this research was conducted in a clinical sample, with objective smartphone tracking, both of which are notoriously difficult to obtain, indicating that the typical sample size limitation is less critical in this context. Second, it should be mentioned that SMS use is now minimal, and we lacked information on messages sent or received via other messaging services like Whatsapp. Third, the operationalization of our smartphone variables, especially passive usage time, remains limited, leaving uncertainty about which exact real-world behaviors they represent and we did not assess hypothesized underlying mechanisms such as emotion regulation, impulsivity, and checking behavior. Further, our data only captured HOW patients used their smartphones and not WHAT they were doing during that time, restricting the interpretability of the behavioral processes underlying the observed associations.

## 5 Conclusion

Excessive smartphone use, particularly high locked screen-time, which might reflect checking behavior could represent a potential digital marker or indicator of outcome in the treatment of BPD. Our findings suggest that individuals with higher dissociative tendencies might be particularly inclined to excessively use their smartphones, potentially as a form of self-regulation or because of difficulties to interrupt related behaviors. In our BPD sample, digital phenotyping offered an efficient and low-threshold way to capture behavioral patterns continuously and in real time, providing early insights into treatment trajectories. In particular, elevated locked screen time was predictive for poorer outcomes and may reflect maladaptive emotion regulation or impulsivity, while the number of incoming calls could represent actual social support processes during therapy. Together, these digital markers hold the potential to enhance treatment monitoring and ultimately improve the clinical management of BPD, though their specificity to BPD needs to be established, and they will require more precise operationalization before reliable implementation in practice. In line with recent calls for responsible digitalization and artificial intelligence use in mental health research, digital phenotyping might complement rather than replace clinical judgment, while also requiring transparency, data privacy, and careful validation of derived interpretations (Beg, 2025).

## Glossary

BPD: Borderline Personality Disorder
BSL-23: Borderline Symptom List
DES-II: Dissociative Experiences Scale
DBT: Dialectical Behavior Therapy
SMS: Short Message Service

## Declaration of Generative AI and AI-assisted technologies in the writing process

This manuscript was enhanced for grammar and readability using OpenAI's ChatGPT. Generative AI was employed to refine language clarity and coherence without altering the scientific content.

## Funding

Ana Macchia received financial support through a doctoral scholarship from the Studienstiftung des deutschen Volkes.

## Declaration of competing interest

The author(s) declare that there were no conflicts of interest with respect to the authorship or the publication of this article.

## Data Availability

The data of this study are publicly available at https://cloudstore.uni-ulm.de/s/FB9ZAzsktTFdeid. The Additional materials, including detailed study procedures and measures, are available in the preregistration https://osf.io/dfq9y/?view_only=4c19b891bb6448009b22f60b2552bd73.
